# Instructional strategies and course design for teaching statistics online: perspectives from online students

**DOI:** 10.1186/s40594-017-0096-x

**Published:** 2017-12-29

**Authors:** Dazhi Yang

**Affiliations:** Boise State University, Boise, Idaho USA

**Keywords:** Instructional strategies, Online course design, Online pedagogy, Statistics, Teaching STEM online, Educational research

## Abstract

**Background:**

Teaching online is a different experience from that of teaching in a face-to-face setting. Knowledge and skills developed for teaching face-to-face classes are not adequate preparation for teaching online. It is even more challenging to teach science, technology, engineering and math (STEM) courses completely online because these courses usually require more hands-on activities and live demonstrations. Although the demand for online STEM courses has never been higher, little has been done to develop effective instructional and online course design strategies for teaching STEM courses online. This paper reports the effectiveness of the instructional strategies adopted and the online course design features in a fully online statistics course from the students’ perspectives. The online statistics course was an introductory, quantitative research course that covered common statistical concepts and focused on the application of educational research concepts for graduate students in educational technology. In terms of the statistics concepts covered, the course was similar to an introductory statistics class for students majoring in science, technology, math and engineering (STEM). The participants were mostly K-20 (meaning from kindergarten to college) instructors who had knowledge of instructional strategies.

**Results:**

Data collected from participants’ reflections and course evaluations revealed that a range of instructional strategies and course design features were effective and helped students learn statistics in an online environment. Specifically, case studies, video demonstrations, instructor’s notes, mini projects, and an online discussion forum were most effective. For online course design features, consistent structure, various resources and learning activities, and the application focused course content were found to be effective.

**Conclusions:**

The implications of this study include effective instructional strategies and online course design for application-oriented STEM courses such as physics and engineering. The study results can be used to guide online teaching and learning as well as online course design for instructors, course designers, and students in STEM fields.

## Background

Online education, including innovative and responsive online course designs, as well as research on student opportunities to interact with online course content continues to grow in higher education. The widespread use of smartphones and mobile applications make online courses appealing to students who conduct coursework remotely, furthering the concept of learning anywhere and anyplace (Smith 2015). The Babson Survey Research Group estimates over 6 million college students are currently enrolled in a distance learning program (Allen and Seaman 2017). At community colleges, the online education growth rate of 4.7% in 2014 was more than the college population growth rate (Smith 2015). A majority of higher education institutions consider online learning as part of their strategic growth (Allen and Seaman 2015). In practice, online courses are increasingly being offered to maximize learning opportunities and reach more students.

The demand for online science, technology, engineering and math (STEM) courses has never been higher, and more STEM instructors are teaching or will be teaching such courses online. However, teaching online is a fundamentally different experience from that of teaching in a face-to-face setting (Davis and Snyder 2012; Juan et al. 2011). Literature suggests that knowledge and skills developed for teaching face-to-face classes are not adequate preparation for teaching online (Deubel 2008). Instructors teaching online often find the experience more difficult and time-consuming (York et al. 2007). It is even more challenging to teach STEM and other quantitatively oriented courses completely online because these courses usually require more hands-on activities and live demonstrations (Akdemir 2010). For example, teaching an online experimental science course is acknowledged to be difficult (Mosse and Wright 2010) due to the required live demonstrations, associated safety concerns, and must-have equipment (e.g., lab supplies). It is also difficult to teach technology, engineering, and math courses completely online (Akdemir 2010). Despite these concerns, little has been done to develop effective instructional and online course design strategies for teaching STEM courses online (Akdemir 2010).

Instructional strategies, which refer to specific methods and approaches that “provide the conditions under which learning goals will most likely be attained” (Driscoll 2000, p. 344), are critical factors impacting online learning and learning experiences (Fresen 2005; Schaller et al. 2002). Few teacher education programs in the USA offer any training in learning theories or teaching pedagogies appropriate for online learning environments (Patrick and Dawley 2009). Most faculty members in higher education “have little or no formal training as teachers” and tend to teach the way they were taught (Perrin 2004, p. 4). Additionally, simple, unaltered adoption of traditional instructional methods while teaching online will not produce the desired outcome (York et al. 2007). Untrained and unprepared STEM instructors are sometimes caught in difficult circumstances when they are tasked to teach an online class. This untenable situation may have a dramatic effect on students’ perceptions of online STEM courses.

Research showed that students’ perceptions of their overall learning experience with instructional strategies and online course design not only affected their perceived learning but also their overall satisfaction of an online course (Myers and Schiltz 2012). Even if there was no significant difference between online and face-to-face statistics courses in terms of learning gains, there was a difference between students’ perceived learning and learning experience (Summers et al. 2005). Although educators agree that math and statistics can be taught online despite their application-based nature (Akdemir 2010), little has been done in developing effective instructional strategies for teaching such courses online (Bonk 2001). In addition, students’ perceptions and attitudes have not been adequately examined when investigating important indicators of successful learning in online statistics courses (Myers and Schiltz 2012).

Most previous studies examining the effectiveness of online statistics courses focused on learning gains (Myers and Schiltz 2012). Student perceptions of the instructional strategies adopted in their online courses, including reflections of the online course design, have not been adequately examined in such contexts. As such, this study focused on examining student feedback and perspectives of the effectiveness of the instructional strategies and online course design features adopted in a completely online statistics course. The purpose of this study was to identify the kinds of online instructional strategies and course designs that effectively helped the students learn statistical concepts.

### Literature review

### Online instructional strategies

Online instructional strategies refer to the methods and approaches that guide the organization of learning activities, course content, and student engagement in online courses (Bonk and Dennen 2003). Posting self-introduction videos at the beginning of an online class so that participants may feel that they know their peers better is an example of an instructional strategy. Despite the lack of available research on effective instructional strategies for teaching STEM courses online, more and more STEM instructors agree that STEM courses can be taught online despite their application-based nature (Akdemir 2010; Summers et al. 2005).

Strategies frequently adopted in online courses include (1) promoting interactivity through asynchronous and synchronous communication or delivery (Ku et al. 2011; Lawton et al. 2012); (2) facilitating the application of concepts (Steinberg 2010; Strang 2012); (3) using video demonstrations, such as screencasts for demonstrating tools and programs (Gemmell et al. 2011); and (4) conveying a strong social presence or a sense of belonging to a learning community (Thomas et al. 2008; Zhang and Walls 2006).

#### Promoting interaction

Students in online quantitative courses such as statistics have limited access to face-to-face support and have a higher attrition rate than that of face-to-face courses (Ariadurai and Manohanthan 2008). The lack of appropriate and deep interaction is a common issue in online courses due to the fact that students and instructors are located in different geological locations (Moore 1991). This lack of interaction can easily result in a sense of isolation and frustration and a high dropout rate in online courses (Willging and Johnson 2004).

Interaction enables students in online classes to be active and collaborative learners. There are three types of interaction in online courses: student-to-instructor, student-to-content, and student-to-student interaction (Moore 1989). Student-to-instructor interaction refers to dialog between students and the instructor, as well as the engagement of the students and instructor in the learning and teaching process (York et al. 2007). Student-to-content interaction refers to the amount of substantive interaction occurring between the learner(s) and the content (e.g., texts, audios, and videos). Student-to-student interaction refers to the dialog and exchanges between and/or among different participants in an online course. These interactions affect not only how students perceive their own learning and the overall educational experience but also the perceived quality of the instruction and learning in an online course (Bonk and Cunningham 1998). Encouraging students to respond to each other and the instructor in ways that demonstrates critical thinking and application of course concepts can also promote higher level cognitive skills (Davis and Snyder 2012; Lawton et al. 2012). Students who engage in collaborative learning can better relate new knowledge to knowledge they already possess and reflect on their own viewpoint and those of others to arrive at a more comprehensive understanding of an issue (Miller and Redman 2010).

Students may interact with one another and their instructor through synchronous or asynchronous communication. Synchronous communication relies on fixed meetings in real-time and allows concerns to be immediately addressed during the learning process (Ku et al. 2011). For example, using Elluminate, a web conferencing program, to deliver a statistics class (i.e., using synchronous communication) was found to be more effective than having a text-based asynchronous communication delivery mode (Myers and Schiltz 2012). Asynchronous communication offers more flexibility for students who prefer to work independently and provides more time for students to reflect on their learning. In asynchronous communication, students work at their own pace and typically interact with each other through threaded discussion boards (Ku et al. 2011). Asynchronous communication also allows students to reflect on readings and what they are learning from others, elaborate on comments, and post thought-provoking questions to encourage others to think further about a topic. This leads students to further develop their own ideas or consider new ways of thinking (Majeski and Stover 2007). However, students may not be satisfied with their instructors’ delayed explanations to their questions, and may not feel that the instructor is approachable, and/or feel at ease in collaborating with their peers with asynchronous communication (Summers et al. 2005). Strang (2012) noted that mathematical-oriented topics are more difficult to learn and teach, and it may be ideal to have both synchronous and asynchronous communication to facilitate hands-on applications and interaction to improve learning. However, considering the reality that many online students seek online courses to avoid fixed meeting times and traveling to campus, it is practical to adopt asynchronous communication for online courses (Huan et al. 2011).

#### Facilitating the application of course concepts

Instructional strategies, such as problem-based learning and case studies, can provide students an opportunity to experiment and share knowledge with their peers online while exploring complex topics and concepts (Steinberg 2010; Strang 2012). The application of concepts to problem solving can increase engagement in comparison to pure theory. This may be especially important in the case of statistical operation-oriented topics for students who are not mathematic majors (Juan et al. 2011; Summers et al. 2005). During course development, instructors should make use of different instructional strategies including live presentations, laboratory tutorials and simulations, discussions, and peer collaboration to support learning activity, exploration, and creation that may help students construct their own statistical knowledge (Juan et al. 2011). Instructors should place emphasis on mathematical applications instead of abstract theory whenever possible and integrate mathematical and statistical software throughout courses to highlight authentic application of concepts, methods and procedures, and collaborative learning to encourage students to play a more proactive role in their learning (Juan et al. 2011). Although these strategies can effectively facilitate application of concepts in online STEM courses, such strategies need careful preparation.

#### Using video demonstrations

Using video demonstrations can greatly enhance teaching statistics online (Ariadurai and Manohanthan 2008; Gemmell et al. 2011). Videos allow physical demonstrations of new software and difficult concepts for students, who may struggle with not only statistics content but also how to use statistical software packages on their own (Al-Asfour 2012). In an online introductory statistics course, videos demonstrations of SPSS for performing certain statistical tests and procedures can be equivalent to hands-on lab sessions where students practice statistical tests or procedures with SPSS. However, videos must be designed to achieve course objectives and contain the right amount of information for students to comprehend (Huan et al. 2011). In order to avoid information overload, the recommended length of effective demonstration videos should be from 3 to 5 min (Miller and Redman 2010). In addition, video speed must be made appropriate to suit the learning process; otherwise, it may increase learners’ anxieties. Based on the above discussion, it is recommended that online instructors should consider create their own video instead of relying primarily on videos found on the Internet.

#### Creating a sense of social presence

Creating a strong sense of social presence or belonging in an online environment is also extremely beneficial for students wrestling with mathematical concepts and procedures (Zhang and Walls 2006). There are different ways to help create a good sense of social presence in online courses. First, perceived instructor’s support may influence online student’s emotions and motivation (Kim and Hodges 2012). Instructor profiles posted on a course website encourage students to realize that they are connecting with real people and have the desired access to instructor support (Huan et al. 2011). Second, the use of humor, encouraging discussion or feedback, and addressing students by name are also helpful (Zhang and Walls 2006) for creating a good sense of social presence. Majeski and Stover (2007) suggest online instructors provide friendly, welcoming posts to keep students up-to-date on course activities. The researchers also suggest instructors should point out areas where improvement may be needed from the students in order to promote interaction within a good learning community. In addition, Majeski and Stover suggest online instructors should identify students new to online learning and e-mail them individually to welcome them to class to promote a good sense of belonging.

In summary, there are instructional strategies for organizing learning activities, course content, and student engagement in online courses. However, most strategies cannot simply be transferred into online courses without modifications. Each of the instructional strategies (methods or approaches) has limitations when being adopted for online courses. To make necessary modifications and adjustments, online STEM instructors need to be informed of online pedagogy research as well as trained in online instruction (Huber and Lowry 2003). To date, little has been done in developing effective instructional strategies and online course design techniques for STEM instructors (Bonk 2001; Yang 2013). As universities, schools, and organizations offer more online courses and online programs, there is an urgent need for research on online pedagogy, especially pedagogical, and discipline-based guidelines for STEM courses that are application-oriented.

### Online course design

Online course design refers to the features that shape the overall structure of the course, including learning activities, sequence of content and communication, and structure of assignments. In most cases, course design drives the instructional strategy adopted in online courses. While some elements of an online course may be predetermined by an institutional template, the presentation and communication of content, resources, and communication preferences or norms can often be structured by the instructor. Institutions may provide outlines or rubrics for instructors to assess their own courses, but instructors make decisions based on the needs of their course. Regardless of instructor competency, content, or student ability, the design of an online course is often among the most powerful factors impacting successful online learning outcomes (Baldwin 2017). The following section discusses the general structure of an online course, which refer to online course design features.

#### Orientation, objectives, and expectations

To design an effective online course, instructors should begin with an organized course orientation including explicit directions including due dates, communication of institutional policies and ethics, and examples of assessments and projects (Robles and Braathen 2002; Song et al. 2004). A well-organized course orientation helps students navigate the course. For the organization of content, clear course learning objectives are critical to help students identify their preparedness for a course, as well as to assist the instructor’s facilitation of student learning (Bozarth et al. 2004). One important component of an online course structure is the conceptual mapping of objectives to assessments, which provides focus for students (Swan et al. 2012).

Students may need time to explore the course components, as well as explicit directions regarding expectations of an online course, such as communication norms (netiquette) and collaborative discussions (Moallem 2003). Moallem recommends that appropriate behaviors and techniques for online discussions—which may be difficult to self-regulate in text-based, asynchronous interactions—should be modeled by the instructor. As one of the primary modes of online course participation, online discussions and interactions are crucial. Many students new to the online setting may not comprehend the time commitment that is required of asynchronous courses and will need to maintain effective time management strategies to avoid falling behind (Bozarth et al. 2004). Students should be expected to know the importance of time management and to make visits to the online course (website) part of a daily routine (Song et al. 2004). Clear expectations from the instructor will assist students in managing their course participation and the assessments.

#### Assessments and engagement

Gaytan and McEwen (2007) suggest that while effective assessments in online courses may vary, students and instructors perceive that the most effective assessments include frequent, formative assignments, as well as projects, portfolios, peer evaluations, and self-assessments. Embedding formative assessment into lessons can assist instructors in evaluating student progress and inform the delivery and design of other instructional plans and assessments (Robles and Braathen 2002). Whenever possible, formative or weekly assignments should deliver immediate information to students (e. g., timed tests and quizzes)—quick, meaningful feedback should be considered among the most crucial communications that benefit both students and instructors (Gaytan and McEwen 2007). Frequent communication between students and their instructors, as well as between students and their peers, promotes student engagement in online courses. In addition, effective communication between and among all participants including the instructor helps to create an environment of variety, spontaneity, and self-directed learning (Ausburn 2004). Examples of effective communication are e-mail reminders, course announcements, and notes in the gradebook from the instructor. In their interactions with students, instructors should consider the social learning experience of the students and seek to become familiar with student learning preferences and concerns (Diaz and Cartnal 1999).

#### Instructional materials and the use of technology

Instructors can control many elements of an online course, including the presentation of materials and the communication of content. Another important element that affects learner experience, however, may reside in the technology and media being utilized in an online course. Ausburn (2004) suggests that a learner’s skills and experience with technology may strongly influence his or her feelings of comfort and security unless an instructor’s reassuring presence is demonstrated. The structure and the technological aspect of an effective online course may be complex. However, course design needs to fit with learner needs and perceptions, encourage technology literacy, contribute to self-efficacy, and provide quality communication, all of which have impact on the experience of students (MacDonald and Thompson 2005). Online instructors should communicate through course design features that facilitate student access and mobility in an online course, which includes easy navigation, legible, user-friendly screen design, and informative multimedia (MacGregor and Lou 2004). The dialog between students and their peers should be developed in many forms––e-mails, chat discussions, synchronous discussions, and even phone calls for complex or imperative needs using an educational technology (Johnson et al. 2000). If students and instructors can acknowledge their shared role as educators and learners in an online course, there are opportunities to not only overcome technical difficulties but also create an effective online learning community (Tisdell et al. 2004).

#### Learner support and accessibility

Supporting the learner through environmental or content difficulties is essential for online course design. Lee et al. (2011) argue that setting up mechanisms and infrastructure to assist students should receive equal consideration to the preparation of the course content. Minimizing issues from the start of the course does not need to be the responsibility of the instructor alone. Institutional resources, such as technical support and tutorials, may be helpful. Providing links or modules that show available course tools or relevant workshop opportunities may also improve student learning experiences with both content and technology (Song et al. 2004).

The design stage of an online course is also the time to prepare for the needs of learners with disabilities (Pearson and Koppi 2002). An online course should be accessible to individuals with physical, mental, and/or emotional disabilities. In addition to making use of institutional supports and the available features of an online course hosting system (e.g., blackboard and Moodle), instructors should seek to add various course contents. For example, course content that is rich in accessible multimedia or assistive capabilities, including interactive examples and simulations, multimedia applications (such as video recordings or synchronous video conferencing), audio transcripts, language translators, and reference books (Bozarth et al. 2004). Weir (2005) suggests that instructors imagine perceiving their online course content through the eyes, ears, and touch of students who are blind, deaf, or physically impaired while developing course materials. Finally, soliciting student feedback or becoming responsive to student difficulties early in the course will allow the online instructor to reconsider the presentation or implementation of course materials.

## Methods

### Research purpose

Since students’ perception of online course design and their learning experience (e.g., sense of presence) affect learning outcomes and satisfaction with a course (Richardson and Swan 2003), it is important to investigate students’ perception and feedback regarding the instructional strategies and course design features in online courses. This study explored effective instructional strategies and course design features in an online statistics class. The specific research question was “What instructional strategies and course design features are perceived to be effective by the students in an online statistic class?”

### Research design

This study intended to examine the effectiveness of the instructional strategies and online course design features from the students’ perspective. The participants selected for the study were mostly K-20 instructors who were teaching full-time, while enrolled in the online course. Their teaching background and knowledge of instructional strategies allowed them to provide unique perspectives and feedback related to effective instructional strategies and course design features that contributed to their learning.

### Context of the study

The statistics course was an online course for students who were pursuing a graduate degree in Educational Technology at a US urban university. The course had no face-to-face meetings and was hosted in Moodle (an online course management system).

This course covered common statistical concepts and their applications in educational research and focused on not only learning statistics concepts but also the application of the concepts in educational research. The subject content level of this course was similar to a statistics course for undergraduate students majoring in STEM fields. Topics covered included the following: (1) understand common statistical concepts, such as hypothesis testing, critical values and *p* values, and confidence interval and their applications in educational research; (2) summarize and describe data according to research questions; (3) input, output, and organize data in SPSS; (4) identify and articulate differences between and among common statistical analysis methods, such as *t* test, chi-square test, and ANOVA; (5) perform and describe descriptive analysis using SPSS; (6) perform and interpret inferential analysis using SPSS; and (7) critique and evaluate common statistical analysis methods in educational research literature.

#### Course setup

The course was divided into seven modules surrounding the seven topics listed above. Each module except the first one lasted more than 1 week, allowing sufficient time for students to complete each module. Instructional strategies adopted in the online course included an online discussion forum, video demonstrations of statistical tests and procedures in SPSS, case studies of published research articles, mini projects, learning reflections, and other module assignments. The online discussion forum was specifically set up for discussing course content-related questions using the standard forum feature in Moodle. There are standard features and functions (e.g., chat room and messages) within Moodle, and the course designer can activate or choose the features according to his or her needs. A chat room was also set up for students to interact with each other and share non-course content-related discussions, such as posting information about a conference or asking for suggestions related to the purchase of a statistical analysis program. Additionally, the instructor provided her own reading notes of the textbook chapters for the students, focusing on the differences and similarities between two or more related concepts or procedures (e.g., *t* tests and ANOVA). The instructor’s notes were word documents and embedded in the online course content in each module.

Students were awarded 10 points (around 6% of total grades) for participation in the online discussions. There were 10 SPSS video demonstrations with a length of 3 to 8 min provided by the instructor. Case studies focused on the students’ articulation and justification of research questions, research methodology, data collection, and data analysis in studies published in educational research. Two research studies (one was a quantitative study and the other a mixed-methods study with a quantitative focus) were provided in modules 4 and 6, respectively, for case studies. There were three mini projects that were decomposed from a typical final project, and each focused on one of the three stages of research: forming a research question, determining appropriate data analysis, and collecting data and conducting the analysis using SPSS. The other module assignments consisted of self-tests (multiple choice questions and brief explanations) and selected questions from the textbook, for example, exercises on calculating confidence intervals. The learning reflection asked students to share their learning experience focusing on the instructional strategies (activities) that helped them learn new concepts in statistics.

### Data collection and analysis

This online course has been offered annually since 2011, first as a temporary and elective course for students in the Master’s program, with a total of more than 80 enrolled students over the past 6 years. The researcher designed and developed the online course and has been teaching it since 2011. The study collected data from the same reflection prompts in 2013, 2014, and 2016, after it became a permanent and required course for the newly established doctoral program in the researcher’s department, following two rounds of offerings. The main structure of the course design and the instructional strategies adopted roughly remained the same. However, the course requirements and its assignments, especially the mini project, were revised for doctoral students. For example, the mini projects required a comprehensive literature review and justification for the research topic that the students proposed to study in their projects. Data were not collected from 2015 due to the researcher’s sabbatical leave.

The reflection questions asked were as follows:

1. What was the most effective instructional strategy (such as videos, case study, and mini-projects) that helped you learn?
2. How do you perceive the instructional strategies adopted or the course design in this online course?
3. Were there any other instructional strategies which were not adopted in this course and you used on your own?
4. Were the learning activities (such as video demonstrations) helpful for you to learn the concepts/materials? What kind of activity (activities) did you find the most helpful?
5. Do you have any other comments for the course? Please be specific.

Participants’ demographic information was also collected from students’ self-introductions. The self-introductions were analyzed so that the participants’ professional backgrounds (teachers or non-teachers) were identified. The reflection data were only collected from the students who were K-20 teachers or instructors. During 2013, students were required to write reflections for each of the six modules and in 2014 and 2016, students were only required to write one final reflection at the end of the course. Reflection data were collected from 39 K-20 teachers or instructors. Each student reflection ranged from one to two single-spaced pages and related to the perceived effect of instructional strategies and online course design. Anonymous, end of semester course evaluation data were also collected.

A deductive approach to code the reflections was adopted (Fereday and Muir-Cochrane 2006). The deductive approach was used because the researcher was able to form a three-code scheme ((1) strategies adopted, (2) strategies not adopted but used by students, (3) course design features) based on her years of teaching the course. During the thematic analysis, the researcher was able to look for patterns and themes regarding learners’ perceived effect of instructional strategies adopted as well as the course design features. The course instructor coded all the reflections. A graduate student also coded five reflections and checked her coding with the course instructor. The two coders achieved more than 95% inter-rater agreement. After the coding, the frequencies were tallied.

## Results

The analysis outcomes are organized around three categories: (1) effective instructional strategies adopted according to the students, (2) strategies not adopted but used by students on their own, and (3) effective online course design features. Specific examples of students’ reflections are also presented to provide insight of students’ perspectives regarding the research question, as well as explanations of the students’ perspectives regarding the emerged categories. Examples provided for each category and its sub-categories were from different participants.

### Effective instructional strategies

**Fig. 1:**
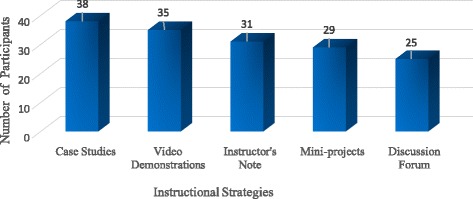
Effective instructional strategies adopted

According to the students, almost all instructional strategies adopted in the online course were helpful. However, case studies were the most effective in helping students learn the statistics concepts and the applications of the concepts in the online course (Fig. 1).

Case studies were perceived to be the most effective and particularly helpful because they allowed students to apply what they learned as well as to connect their learning in a meaningful way. For example, one student reflected,

“… the case study was a great way to apply our new skills to something real-life. I’ve read so many research articles in the past, but I’ve never quite understood the technical side of the analysis. I thought that …seeing a real-life application in a research study helped to put the pieces together – mostly.”

Another student also wrote about how the case studies connected their learning with the applications of what they had learned:

“I enjoyed completing the case study. I think that these [case studies] assignments allow me to apply what I am learning in class in a meaningful way; a way that contributes to my further growth as a researcher and as a reader of research. … It reminds me that teaching using the context in which the knowledge would be used later was very powerful.”

Case studies were also able to help students achieve a deeper understanding of the newly learned concepts. For example, one student reflected:

“They [case studies] force me to work through the details to the point at which I have an in-depth understanding of the concept and can apply it to an actual situation. Answering the cases questions required me to analyze how the research was performed. … The case studies helped me think critically, especially by thinking and responding to the questions…”

The case studies also provided an opportunity for students to compare their learning and understanding with those of their instructor’s and their peers’ through instructor’s feedback and class discussions. In this sense, case studies were an effective instructional strategy for providing opportunity for self-reflective learning that leads to deeper understanding (Penn et al. 2016). For example, one student wrote:

“Case studies have been the most important part of the course for me. I like the problem solving, the sense of a whole and the feeling of acquiring a skill. Most importantly they provided the perfect opportunities to compare my understanding with my professor’s and my peers’ understanding of the same set of questions.”

Following the case studies, video demonstrations of statistical tests and procedures using SPSS were most helpful and effective instructional strategy in the online statistics class. The video demonstrations successfully achieved their intended purpose, teaching students how to perform different statistical procedures in SPSS. It seemed that those video demonstrations effectively replaced a face-to-face, hands-on lab session. For example, a student wrote, “The videos were a life saver with using SPSS. Being able to follow along with someone as they performed all the steps made it much easier to understand this very intimidating program.” In addition, most students agreed that watching a video is much more effective than learning from reading the textbook or a manual for SPSS. For example, one student pointed out: “In most cases, watching a 10-minute video gave me a better understanding than reading a whole chapter in the book.” Interestingly, video demonstrations seemed to be more effective than reading step-by-step instructions and also watching a live demonstration because students could watch recorded demonstrations as much as needed. For example, one student reflected how the video demonstrations helped him,

“I found the video tutorials helpful. I re-viewed them several times to strengthen my understanding. It was nice that the narratives did not just explain what to do, but also why we were doing it and how to interpret the results [output].”

Some students also found that the video demonstrations provided a less intimidating way for them to learn the statistical program of SPSS. This may be due to the fact that students could watch the video demonstrations as much as they needed without worrying about the peer pressure of appearing to fall behind, a situation students might encounter in a face-to-face class. For example, one student reflected:

“The most informative and useful instructional strategy was the videos. As I have gone through this course, I am beginning to look forward to using SPSS. It is not so intimidating to me at this time, which is in large part due to the video demonstrations. I could watch the videos as much as needed just by myself without feeling of dragging down my peers.”

The instructor’s notes emphasizing the key concepts in each chapter and outlining the similarities and differences between and among similar concepts were found to be the third most effective strategy adopted in the course. The instructor’s notes helped students understand key concepts better. For example, one student reflected, “The instructor’s notes were the most helpful for enhancing understanding and emphasizing key points from the book.” Similarly, another student wrote, “Again for me the instructional strategies presented that helped me the most, were the instructional [instructor] notes. The highlighting of core concepts, with clear explanations, aided me in the reading of many chapters...” Some students even gave specific example of where the instructor’s notes helped them the most. For example, one student reflected, “I was struggling to thoroughly understand the difference between the paired and two sample t-tests. The instructor’s notes, particularly, in Chapter 7, were very helpful for understanding the two concepts.”

The instructor’s notes also provided a great guide and acted as an advance organizer (Mayer 1979) for students when they started to read the textbook to decide what content was the most important. For example, one student reflected:

“The instructor’s notes helped me a lot. The textbook is good but wordy and I could read it and read it again and still not be able to figure out what was important and what wasn’t. With the instructor’s notes, I could figure out what was important and let go of things that were unimportant which I did not understand fully.”

Some students found that it was even more effective to combine the instructor’s notes with other available resources, such as course assignments. For example, one student described how he used instructor’s notes,

“The instructional strategies that I found most effective were the videos and instructor notes/summaries. During each module I would read the instructor notes, print the assignments, and refer to each often while reading the assigned textbook chapters. Having access to each resource at once was the most helpful in understanding a concept.”

Based on the students’ reflections, instructor’s notes that focused on the similarities and differences of similar concepts were not only necessary but also one of the most valuable instructional strategies that helped students learn the content in an online statistics class.

The mini projects were several small projects that built into a final project throughout the semester based on the progression of the course content. The mini projects enabled the re-visiting of the same project over the semester and were the fourth most effective instructional strategy adopted. The mini projects served as a valuable building process of the final project and provided students sufficient time and opportunities to digest concepts before applying them. They also allowed the instructor an opportunity to provide focused comments and feedback on specific aspects of the final project while the relevant concepts were under discussion. For example, one student reflected,

“The mini-projects helped me the most in the course because they helped me complete the final project in a much better way, quality wise. I really appreciated the feedback that I received on my first mini-project, and I believe that my next two were much more refined and [more] complete projects due to the feedback received previously.”

Some students also commented that the mini projects provided them the opportunity to experience how a research study worked from beginning to end in a less intimidating way. For example, one student reflected:

“I also liked that we built upon our mini-projects throughout the semester, which was the most effective way to complete a final project. It felt so much less intimidating to be able to chunk it out and work on it piece by piece as we gained new knowledge and insight [about research].”

It seems that mini projects were very effective to teach educational research and the applications of statistics concepts. They also helped students complete a final project that involved different building processes and acquiring the knowledge for building a final project in a progressive way.

Nearly two-thirds (25/39) of the participants considered the online discussion forum to be an effective instructional strategy adopted in the online statistics class. The online discussion forum provided a means for students to seek clarification and answers to their questions and also a way to validate their understanding and promote self-reflective learning. For example, one student wrote:

“Being that this is a reflection … I feel that the discussion forum was the most useful. I was very engaged and was glad that I could not only help other students to problem solve why they were getting an error message when doing their Tukey test but that I understand the concepts being presented. It also validated for me that I am coming to a greater understanding in the class as a whole.”

By contributing to the class via responding to their peers’ questions, most students felt that the online discussion forum was helpful for encouraging peer-to-peer and instructor-to-student interaction. For example, one student reflected, “As most of us are social people and we feel the need to connect with each other through our courses, it is nice to talk with each other, and discuss concepts and ask questions via the online discussions even in this statistics class.”

In addition, online discussions also provided some extra motivation and promoted a healthy competition among peers. For example, one student shared:

“I read a forum post from someone about getting all the research situations correct from Chapter 13. That was all it took to motivate me to try to meet his high standard. I’m happy to say I met that challenge and tied him in his score.”

For those students who did not actively participate in the online discussion forum, they also benefited from having a place to review relevant discussions on course content and check their own understanding via self-reflection. One student reflected:

“The null hypothesis is a difficult concept. By the time I came up with questions, I noticed many had been posed on the discussion board. It was more helpful to me to read the comments of others than to interject a similar question. Eventually, I came to understand it.”

It appeared that an online discussion forum was very necessary. It helped the students discuss course-related problems and provided a way for students to check their understanding and learning against that of their peers.

### Other strategies students used on their own

#### Using outside online resources

Other instructional strategies that were helpful for promoting students conceptual understanding of the statistical content were searching the Internet and using outside relevant resources that were not provided by the instructor. The majority of the students (35/39) used outside resources, such as websites, online videos, and even people to help their learning. The most frequently used outside resources included the Google search engine; the Khan Academy; Research Gate’s Q&A section; iTunes U; and textbooks, such as textbooks on Amazon called *Activity-based Statistics* and *The Cartoon Guide to Statistics*.

Some students referred to outside relevant websites when they could not easily make full sense of the required textbook. In fact, more than half of the students mentioned using Google search or external websites and resources in their reflections. The extra research for relevant resources of the course content and reading additional information online or even other textbooks seemed to be one of the most helpful instructional strategies for the students in this online course. For example, one student reflected: “I fully expect to find my own information in many courses, so this was not a concern for me. I do think the textbook was difficult to learn from, which may be why I looked for more outside resources.”

Relevant outside websites, including videos and interpretations of SPSS outputs helped clarify difficult topics and also served to alleviate students’ fear and anxiety towards learning statistics. Some students shared extra resources they found with their peers in the online discussion forum. For example, one student wrote:

“I set up a Diigo site for posting good stats sites and shared the site with my classmates. …I spent a great deal [of time] doing research on the Internet to clarify concepts when I was still uncertain about them, especially for the t-tests.”

However, the use of outside resources may be due to the students’ different backgrounds and/or entry knowledge levels. For example, one student used outside resources because the textbook was difficult for her to understand. Another more advanced student used outside resources due to the lack of depth of the mathematical concepts covered. For example, the more knowledgeable student reflected: “The textbook overlooked the mathematical concepts that underlie the different [statistics] concepts. After reading the text, I relied on online resources from other universities to better understand the mathematical concepts.”

As a result of different backgrounds and the access to different resources, students used a variety of resources. However, the students experienced challenges finding and using outside resources. One challenge was to find the right resource on the exact topic. For example, one student wrote, “The challenge for me was finding the right video about the exact topic I was looking for.” Another challenge was not all resources exactly met the students’ needs. For example, one student wrote, “I think some of the videos [found on the Internet] were focused on “do this” and didn’t add to my understanding of why I was doing what I was doing.” The third challenge was that different sources may use different terminologies for the same or similar concepts. For example, one student reported, “I have found myself using Google to better understand some concepts. However, sometimes, different terms or names have been used by different sources than the terms used by our textbook or instructor. This sometime caused more confusion.”

#### Practicing textbook examples and chapter problems

More than half of the students (21/39) also tried to figure out the statistical or mathematical process underlying a statistics procedure on their own in order to achieve a better understanding by working out the textbook examples and/or chapter exercises. For example, some students calculated the confidence interval example in the textbook using a pencil and a piece of paper. Working out the textbook examples on their own helped some students understand the concepts better. For example, one student reflected, “I find calculating the statistics by hand gives me a deeper understanding of the statistics since I am forced to work through the data and the resulting variables.” Similarly, working out the chapter problems and exercises on their own helped or reinforced students’ understanding of the statistics concepts or procedures. One student wrote:

“For many of the chapters, I worked most, if not all, of the practice problems at the end of each chapter. Working the practice problems at the end of the chapters helped me to execute and reinforce the statistical concepts presented. Although these were not assigned, I felt as if figuring the statistics by hand often helped me to comprehend the important concepts in the chapters.”

Given more time, more students might have been able and/or willing to work out the textbook examples and the end of chapter practice exercises.

#### Seeking help from peers, colleagues, and friends

Some students (15/39) also sought out people who were knowledgeable about statistics to help their learning in this online course. For example, one student reflected:

“I have also been working with two other students in the class. Alex and Paul [pseudonyms] have helped me tremendously and I don’t think I would have been able to finish this class without their support. Alex especially helped me to gain the confidence I needed to move forward. … I am very grateful that I didn’t give up.”

Students sought help from various people, including their spouses, friends, and colleagues. For example, one student wrote, “I also sought assistance from two colleagues: A statistics teacher … and a principal who recently completed a doctoral program.” This finding made the researcher wonder if students in a face-to-face class would seek out help from knowledgeable people the same way as those in an online course.

#### One-to-one phone calls

In addition to e-mails asking for clarifications of course content and weekly assignments, some students (9/39) also contacted the instructor for one-to-one phone calls to help clarify some concepts, such as the dependent vs. independent variables.

It seemed that the majority of students in the online class actively engaged in learning, which was revealed in their thoughtful reflections. The students completed the course tasks assigned by the instructor and also actively sought out extra resources and help during their learning process. Although the outside resources provided students with multiple opportunities to interact and learn the course content, it was not without challenge. Online course instructors need to be conscious of this aspect when designing an online course.

### Effective online course design features

For this aspect, the researcher first examined the end of semester course evaluations, specifically the four questions related to most important course structure and learning activities as anonymously reported by 40 students (Table 1)*.* The four course evaluation questions presented in Table 1 related to clear objectives and the alignment between course assessment and objectives and organizations of learning activities (peer collaboration/learning community) in online courses*.* The course design features covered by the four questions were also determined by the course instructor/designer. Table 1 lists the average means of students’ responses to the four course design questions on a one to five Likert scale with five being the highest score.

**Table 1:** Student evaluations of course design features by semester/year

Questions	2013 average	2014 average	2016 average	Weighted average
1. Assessments and/or other products reflect course objectives.	4.25	4.23	4.42	4.30
2. The readings were aligned with course objectives.	4.33	4.40	4.67	4.47
3. The objectives of the course were clearly explained.	4.25	4.27	4.41	4.31
4. Peer collaboration was offered.	3.79	4.00	3.89	3.88
Number of students responded (anonymously).	12	15	13	13

Note: The total number of participants over the 3 years in Table 1 is 40

Based on the course evaluations, the course objectives were clear and the assessments and course materials were well aligned with course objectives because the weighted averages of students’ responses to the first three questions were 4.3 or more on a scale of one to five. Students’ responses to the peer collaboration question were much lower than those to the other three questions, which again demonstrates the challenge of creating an effective and collaborative learning community in an online STEM course (Bacon and MacKinnon 2016).

Next, the researcher coded the 39 reflections for effective course design features. Overall, the students considered the course well designed. Students identified the following course features as being helpful in this course:

#### Consistent structure of the course

The majority of the students (31/39) viewed the consistent structure and layout of each module’s content as helpful and effective. The consistent structure or layout of each module helped students get familiar with the course as well as ease their fears of statistics. For example, one student wrote:

“I enjoyed the consistency of the course structure. The first module seemed quite difficult and there was a lot content, but because the following modules were very similar I eventually got the hang of things and felt much more comfortable and confident as I knew what was to expect.”

This shows that a consistent course layout with friendly navigation within an online course is very important to keep students oriented *and* reduce the fear of a topic that is perceived as challenging.

#### Various resources, assignments, and activities

Some students (28/39) also liked the overall course design because it offered a variety of resources, assignments, and activities. All assignments and activities organized in the course worked cohesively to help students obtain the learning objectives. This aspect was also reflected in the students’ course evaluations regarding the good alignment between course objectives, materials, and assessments. For example, one student reflected:

“I think this class was set up very well. I think the pace, resources, and lessons all worked together cohesively and helped assuage my fears. I appreciated the instructor’s notes throughout each module (what to pay close attention to and what to skim) …. Considering the complexity of the content I could have fallen behind, but the class was set up to provide multiple resources/assignments to study the content and allow us to apply what we learned.”

Similarly, one student wrote, “I enjoyed the flow of the course. The balance between the bookwork [selected chapter exercises], the SPSS exercises, and external assignments [such as case studies] was good.” It seemed that providing different resources, assignments, and activities in an online course can create a combination of ways for students to learn as well as to check if they understood the materials correctly.

#### The application focus

Some students (27/39) enjoyed the application focus of the course in an introductory statistics course on educational research. For example, one student wrote, “I appreciate the way this course was structured. We had opportunities to be introduced to the material, engage one another in conversation, and then apply our knowledge.” Similarly, another student wrote, “I thoroughly enjoyed the applied statistics. The topic has made me think about things outside of school, especially politics and news reporting of polls.” In regard to how this course was set up differently from a similar course for STEM majors, one student’s quote provided a perfect explanation, “The case studies and mini-projects were very effective for shifting the understanding of concepts into practice. These added an unexpected dimension [application] to what I anticipated to be a basic math course. I appreciated this addition to the coursework.”

## Discussions and conclusion

According to the students’ reflections, instructional strategies such as case studies, video demonstration, instructor’s notes, mini projects, and discussion forums were among the most effective instructional strategies. Case studies were perceived to be the most effective instructional strategy in the applied statistics class. This is not surprising considering the application focus of the online statistics class and the virtues of case studies as an instructional strategy, such as bridging “the gap between theory and practice” (Barkley et al. 2005, p. 182) and allowing students to identify the problem and argue different perspectives.

The specific reason why students perceived case studies to be the most helpful strategy in this online class was that case studies use existing studies as a reference to help students connect their learning and build their own knowledge on all aspects of research including data analysis. Case studies also provided context for the use of specific methods that helped create a framework from which to scaffold learning. The use of case studies is also a great way to allow students to see how others apply statistical concepts. It was a valuable exercise to look at the published research of others and identify how the concepts the students were studying were actually applied in an authentic manner. However, as effective as case studies can be, instructors are recommended to use other forms of assignments or activities to allow students to research and explore different aspects of their learning since case studies provide generally similar information or issues that not all students may find interesting.

Due to the need of live demonstrations of SPSS software in the online class, video demonstrations were necessary and effective in helping students learn how to use and interpret the outputs of SPSS. For video demonstrations to be more effective, videos need to focus on both the step-by-step “how to” procedures and also the meaning of the outputs related to the concepts in the specific course. This is why instructors still need to create their own videos from time to time despite the fact that there are many videos readily available online. This sentiment was echoed in the students’ reflections by the challenge of using outside resources, and the difficulty of finding the exact videos needed, on their own.

Interestingly enough, the instructor’s notes were found to be the third most effective strategy in this course. Almost all instructors would provide course materials in some forms, such as in PowerPoint for students in online classes as class notes. However, based on the students’ reflections, effective instructor’s notes should focus on the similarities and differences between and among similar concepts, pointing out what to look for and providing hints of what parts of the textbook can be skimmed.

There are different ways to structure the final project in online courses. Mini projects in this course were found to be one of the most effective strategies to complete the final project. Mini projects allowed students sufficient time to master particular concepts and skills, such as checking initial data and forming a research question while internalizing the learning. Mini projects provided a real-world application for the course and added value and meaning throughout the course work for students.

Some instructional strategies and activities even achieved more than their initial objectives. For example, the online discussion forum was initially set up to provide a means for students to discuss course-related questions, an informal type of peer collaboration. In fact, the online discussion provided a place for students to post their questions and issues and also helped promote a sense of social presence and a sense of contributing to the class by providing responses to peers’ questions and sharing helpful resources. However, from the student course evaluation data, the students did not seem to consider the online discussions as peer collaboration since their responses to peer collaboration clearly lagged behind other aspects of the course design. It is clear, however, that online discussions are important in an online statistics class. This study also confirmed the challenge to design and promote peer collaboration and teamwork in online courses (Bacon and MacKinnon 2016). STEM instructors need to keep in mind that “online learning is as much a social activity as an individual one” (Brindley et al. 2009, p. 1). The researcher would recommend STEM instructors and course designers work with or at least consult with people who have expertise in instructional strategies when choosing and implementing instructional strategies in online courses.

As for those instructional strategies used by students on their own, it was not a surprise to see that most of the students searched and used outside resources. This is to be expected from any students who are proficient computer users. However, as online course instructors, we need to take this aspect into consideration and be aware of possible challenges faced by the students when using outside online resources while designing and organizing our course. It was interesting to find out how often students rely on outside online resources to learn and clarify course materials. It would be helpful to direct the students on how to evaluate online resources, such as checking the credibility and qualifications of the authors and sources of the online resources. After all, not all information on the Internet are correct or valid, which is especially true for technical STEM subjects like statistics. One way to achieve this purpose is to use a social bookmarking website tool such as Diigo for posting relevant websites with quick comments and notes. Online instructors may need to do some searching and place filters or warnings on some search outcomes, if possible.

Future research on how students find and rate outside materials relative to their online courses is recommended. In addition, online instructors should specifically encourage students to work out textbook examples and the end of chapter exercises as much as possible, as well as seek help from their peers, colleagues, and knowledgeable friends. Engaging in one-to-one discussions with the course instructor, a strategy that could be included in the course syllabus, is also recommended. A phone call may help clear up a lot of confusion. Thus, for online courses such as this, offering one or two pre-scheduled virtual meet-ups so the entire class could connect with each other could be helpful. Future research on how students collaboratively extend and build a course using the online resources they discover and what best supports this collaborative course building is also recommended. Last but not the least, research on the use of different types of case studies and their effectiveness in an online course is necessary.

Effective online course design features included clear course objectives, good alignment between course objectives and assessments, consistent module structure, a variety of assignments and learning activities, and a good balance between theory and applications. For this course, it was critical to have a good balance of introducing the concepts and the applications of the concepts. It is always a challenge to achieve both in one class because applying the learning also takes time. However, students seemed to have enjoyed the application aspect of the course as long as they were given a sufficient amount of time to process their learning before they applied the knowledge.

It is apparent that different instructional strategies and course design features had different impact. Thus, online STEM course designers should consider multiple instructional strategies and course design features to promote the learning of course content, including the development of a learning community, and the means for more knowledgeable students to teach and share their expertise and resources with peers. The application aspect of this study focused on educational research. However, these strategies could be appropriate to other kinds of applications, such as experimental design and data analysis in industry.

Limitations of this study mainly consist of the following aspects. First, the students enrolled in this course were competent in technology, in terms of retrieving and locating online resources since they had already taken several online courses prior to enrolling in this course. Students with appropriate knowledge and skills with technology may tend to rely more on the Internet for supplemental resources that can help them learn new concepts. Second, due to the nature of this course, applied statistics for educational research, the students may have used more websites and outside resources as a way to supplement their textbook and course materials. Similar studies with different course topics focusing on effective instructional strategies are needed.
